# Pharmacists’ attitudes towards interprofessional collaboration to optimise medication use in older patients in Switzerland: a survey study

**DOI:** 10.1186/s12913-024-11339-8

**Published:** 2024-07-26

**Authors:** Renata Vidonscky Lüthold, Damien Cateau, Stephen Philip Jenkinson, Sven Streit, Katharina Tabea Jungo

**Affiliations:** 1 Institute of Primary Health Care (BIHAM), University of Bern, 3012 Bern, Switzerland; 2 Graduate School for Health Sciences, University of Bern, 3012 Bern, Switzerland; 3 Centre for Primary Care and Public Health (Unisanté), University of Lausanne, 1015 Lausanne, Switzerland; 4 Division of Pharmacoepidemiology and Pharmacoeconomics and Center for Healthcare Delivery Sciences (C4HDS), Department of Medicine, Brigham and Women’s Hospital and Harvard Medical School, 02115 MA Boston, USA

**Keywords:** Polypharmacy, Deprescribing, Medication optimization, Medication review, Interprofessional collaboration, Pharmacists, Older adults

## Abstract

**Background:**

Collaboration between physicians and pharmacists facilitates the conduct of medication optimisation efforts. In the context of deprescribing, pharmacists’ roles are often described as making deprescribing recommendations to physicians. Little is known about factors associated with pharmacists’ willingness to make deprescribing recommendations and their interprofessional collaboration with physicians in Swiss primary care settings.

**Objective:**

To explore pharmacists' perspectives on medication optimisation and deprescribing in older adults, and their preferences for interprofessional collaboration in Swiss primary care settings.

**Methods:**

In this cross-sectional study, a random sample of 1000 pharmacist members of the Swiss Pharmacists Association pharmaSuisse was invited to participate in a survey on medication optimisation, deprescribing, and interprofessional collaboration. The survey contained three case vignettes of multimorbid patients with polypharmacy aged ≥ 80 years old, with different levels of dependency in activities in daily living (ADL) and cardiovascular disease (CVD). For each case vignette, pharmacists were asked if and which medications they would deprescribe. We calculated proportions of pharmacists’ willingness to deprescribe by case vignette and performed a multilevel logistic regression to assess associations between CVD, ADL, and willingness to deprescribe.

**Results:**

One hundred thirty-eight (14%) pharmacists responded to the survey: 113 (82%) were female, their mean age was 44 years (*SD* = 11), and 66% (*n* = 77) reported having never received any specific training on how to conduct structured medication reviews. Eighty-three (72%) pharmacists reported to be confident in identifying deprescribing opportunities. All pharmacists were willing to deprescribe ≥ 1 medication in all vignettes. Patients with CVD were at lower odds of having medications deprescribed (OR = 0.27, 95%CI 0.21 to 0.36). Willingness to deprescribe was lower with higher dependency in ADL (medium versus low dependency: OR = 0.68, 95%CI 0.54 to 0.87, high versus low dependency: OR = 0.72, 95%CI 0.56 to 0.91). However, the effect of dependency in ADL on willingness to deprescribe was significantly modified by the history of CVD. One hundred five pharmacists (97%) reported to interact with physicians to clarify questions regarding prescriptions at least once a week and 88 (81%) wished to be more involved in deprescribing and medication review.

**Conclusion:**

Pharmacists were willing to make deprescribing suggestions for older patients with polypharmacy, but two-thirds reported having received no formal training on how to perform structured medication reviews. Pharmacists would like to be more involved in the process of medication review and deprescribing, which should be leveraged in the context of Swiss primary care settings.

**Supplementary Information:**

The online version contains supplementary material available at 10.1186/s12913-024-11339-8.

## Background

The worldwide ageing population has been leading to new challenges in the health care of older adults. With ageing, older adults are more susceptible to having multiple diseases (known as multimorbidity), which often leads to polypharmacy (commonly defined as the regular use of ≥ 5 medications) [1–3]. When polypharmacy involves medications without a clinical indication, in too high doses, or medications for which harms outweigh potential benefits, it is commonly referred to as inappropriate polypharmacy, which is common among older adults [3–7]. Due to the age-related pharmacokinetics and pharmacodynamics changes in the body, older adults are at high risk for adverse events led by inappropriate polypharmacy [5, 8]. Inappropriate polypharmacy has been associated with several health issues, such as an increased fall risk, cognitive decline, and adverse drug reactions [8–10]. Studies have shown that many older adults are receiving medications without an indication, in too high doses, or for too long (overprescribing) or are not receiving the appropriate treatment (underprescribing) [11, 12]. To address over- and underprescribing medication reviews and deprescribing (stopping or reducing medications for which risks outweigh benefits) should be part of patient care [13–15].

Older adults with multimorbidity and polypharmacy commonly see different healthcare providers due to their complex healthcare needs. To optimise older adults’ medication use, collaboration among health professionals is crucial [16, 17]. Pharmacists are healthcare professionals who are in constant contact with patients, they have excellent knowledge about medications, and therefore they are equipped to play a key role in deprescribing and medication optimisation [18–21]. The collaboration between pharmacists and general practitioners (GPs) is promising for the conduct of medication optimisation efforts [19, 22]. Several studies have shown that a multidisciplinary intervention, including pharmacists, had a positive impact on deprescribing in long-term care facilities [23–26] and facilitated deprescribing in primary care settings [19, 22, 27].

In these interprofessional collaborations, the role of pharmacists is often described as making deprescribing recommendations to physicians and proposing treatment plan modifications. A qualitative study in nursing homes conducted in the French-speaking part of Switzerland aimed at informing future intervention in nursing homes and found that pharmacists seemed to be more willing to put deprescribing into practice, while nurses and physicians were more cautious [28]. Studies in other countries, however, reported pharmacists to be less willing to deprescribe medications compared to physicians [29, 30]. Despite the promising involvement of pharmacists in medication optimisation, there are also many barriers to effective interprofessional collaborations [29–31]. For instance, pharmacists are sometimes hesitant to make recommendations to physicians due to the fear of jeopardising their collaboration and they fear that their recommendations could be perceived as inappropriate [31]. Lack of access to the patients’ health information has also been reported as a barrier for interprofessional collaboration [29, 32]. These barriers are likely also true in the context of Swiss primary care settings. In Switzerland and worldwide, the role of pharmacists in primary care has been changing with their activities becoming more clinical and patient-focused [33]. Community pharmacists in Switzerland provide some services aiming to optimise medication use (e.g., ‘polymedication check’ (an ‘intermediate’ medication review and screening for adherence issues for patients using minimum three prescription medications), preparation of ‘weekly pill organizer’, directly observed therapy [33, 34]), and work collaboratively with physicians to perform medication reviews in nursing homes [33–35]. In addition, community pharmacists are engaged in physician-pharmacist quality circles [36]. The aim of this interprofessional collaboration is to optimise prescription habits by discussing the newest evidence-based guidelines, sharing knowledge, and discussing case studies [37, 38]. However, little is known about interprofessional collaboration for optimising medications in Swiss primary care settings.

In this survey study, we aimed i) to explore the current practices of pharmacists working in Switzerland related to conducting medication reviews, ii) to understand pharmacists’ attitudes towards making deprescribing recommendations in adults ≥ 80 years with polypharmacy and how patients’ history of cardiovascular disease (CVD) and dependency in activities of daily living (ADL) are associated with pharmacists' willingness to make deprescribing recommendations, and iii) to explore pharmacists’ experiences with and wishes for interprofessional collaboration between pharmacists and physicians with regards to medication optimisation.

## Methods

### Study design and data collection

In this cross-sectional survey study, a random sample of 1000 pharmacist members of the Swiss Pharmacists Association (pharmaSuisse) were invited to participate in an online survey. Participants were invited in two batches of 500 each. The first batch received a reminder and the second one received one email. Data was collected between June and December 2023. The questionnaire was available in German and French on SurveyMonkey [39] (for the English translation see Additional file 1). The questionnaire was anonymous, and pharmacists did not receive any compensation.

### Inclusion criteria

Inclusion criteria were to work as a pharmacist in a community pharmacy, hospital, nursing home or home care in Switzerland, and to be an active member of pharmaSuisse. Pharmacists working in other settings (e.g., industry) were excluded because we were interested in pharmacists with direct patient contact.

### Questionnaire

Seven pharmacists piloted the survey before the start of the data collection. The questionnaire contained 41 questions regarding pharmacists' sociodemographic characteristics and work settings, familiarity, and experiences with deprescribing and medication reviews, and their interprofessional collaboration with physicians. In our questionnaire, *medication review* was defined as “*a structured evaluation of a patients’ medications, including identifying medication-related problems and making concrete suggestions for improvement. The aim of a medication review is to identify, solve and prevent drug-related problems to optimise drug therapy, reduce drug side effects and improve clinical outcomes*” (adapted from [40]). To assess pharmacists’ experiences with medication reviews, we adapted the *Tool for Assessing Ambulatory Care Pharmacist Practice* (TAAPP) [41]. To assess confidence in deprescribing, we used the confidence scale from Heinrich et al. [18]. To assess pharmacists’ experiences with and wishes for interprofessional collaboration, we adapted the questions from the *Physician/Pharmacist Collaboration Index* (PPCI) so that they addressed collaboration with physicians in general and not one specific physician [42]. The adapted score from the PPCI ranged from 10 to 70, with higher scores indicating greater collaboration. Next, we presented three case vignettes describing hypothetical patients aged ≥ 80 years with polypharmacy to the pharmacists to assess their willingness to deprescribe. We adapted the case vignettes from the study conducted by Jungo et al. with general practitioners in 31 countries [43]. Hypothetical patients in the case vignettes differed in terms of dependency in activities of daily living (ADL) and history of cardiovascular disease (CVD) (Additional file 1, part E). Case 1 presented a patient with low dependency in ADL, case 2, medium dependency, and case 3 high dependency. Each case vignette was first presented without a CVD and later after a cardiovascular event. Pharmacists were asked if and why they would stop or reduce any medication in each case vignette.

### Sample size calculation

We used the power one proportion function in Stata to calculate the sample size. Based on the 74% of pharmacists found to be confident to discuss deprescribing interventions [18], we would need to recruit 106 pharmacists in Switzerland at a power 0.80 to reach an effect size of 0.1 to detect a difference in the proportion of pharmacists who are confident to discuss deprescribing interventions. To account for potential missing data, we considered that around 20% of respondents would not complete the entire survey and verified that 3% of pharmacist members of pharmaSuisse were not working in an eligible setting. The minimum sample size was therefore 137 pharmacists.

### Statistical analysis

We used descriptive statistics to report pharmacists’ characteristics. Continuous variables were presented as means and standard deviations and categorical variables as frequencies and percentages. We used two-sample test of proportions to compare the percentages of deprescribing recommendations across the case vignettes. We performed a multilevel logistic regression at the medication level to assess the association between the willingness to deprescribe and pharmacists’ and patient characteristics (CVD, dependency in ADL). After noticing differences in willingness to deprescribe by level of dependency in ADL, we performed an additional sensitivity analysis at the level of CVD to further understand the relationship between willingness to deprescribe and dependency in ADL. Covariables included in the model were selected based on clinical rationale: age, gender, specialization training in community pharmacy, frequency of interaction with older adults, and having obtained a specific training on how to perform structured medication reviews. We identified the data to be missing at random and used a complete case analysis method. Analyses were performed with Stata 16.1 [44]. A two-sided p-value of 0.05 was considered statistically significant. Free text responses were assigned to not pre-defined categories.

### Ethical approval

This study did not fall within the scope of the Swiss Human Research act and therefore a waiver of non-responsibility was obtained from the competent ethics committee of the canton of Bern (Req-2021–01101).

## Results

### Sociodemographic characteristics

Of the 1000 pharmacists invited to respond to the survey, 138 (14%) pharmacists accepted to participate in our study. 113 (82%) were female, with a mean age of 44 years old (*SD* = 11), and a mean of 18 (*SD* = 11) years working as a pharmacist (Table 1). Regarding further training, 43 (31%) pharmacists had a specialist training (FPH) in community pharmacy and 57 (41%) a specific training (FPH) in anamneses in primary care. 132 (96%) of the pharmacists worked in a community pharmacy or pharmacy combined with drugstore. Pharmacists were working in 20 out 26 cantons in the different language regions of Switzerland (Additional file 2). Pharmacists reported that on average 40% (*SD* = 22) of their daily patients are ≥ 70 years old and have polypharmacy.

**Table 1:** Case vignettes overview

**Dependency in activities of daily living (ADL)**	***Vignette 1 – Low dependency in ADL:*** Male, 82 years old, lives in his own home with his wife, good physical and cognitive condition, prepares his own medication, does household tasks and daily activities independently and does not need any help
	***Vignette 2 – Medium dependency in ADL:*** Male, 82 years old, lives in his own home with his wife, increasing forgetfulness, unable to do household tasks and needs help from third parties for personal hygiene, getting dressed/undressed and preparing medication
	***Vignette 3 – High dependency in ADL*:** Male, 82 years old, lives with his wife in a nursing home, uses a walker, needs help with personal care, cognitive impairment, unintended weight loss
**Daily medication**	**Identical medication list for all case vignettes:** ▪ Aspirin 100 mg once daily ▪ Atorvastatin 40 mg once daily ▪ Enalapril 10 mg once daily ▪ Amlodipine 5 mg once daily ▪ Paracetamol 1 g three times a day ▪ Tramadol 50 mg twice daily ▪ Pantoprazole 20 mg once daily
**Medical history: other diagnoses**	**Each case vignette was presented twice (Part 1 and Part 2):** ▪ Chronic back pain ▪ Non-smoking ▪ Hypertension ▪ Dyslipidemia ▪ **Vignette—Part 1:** No history of cardiovascular disease ▪ **Vignette—Part 2:** History of cardiovascular disease

### Attitudes towards medication review and deprescribing

Current practices of pharmacists working in Switzerland related to conducting medication reviews and attitudes towards deprescribing are shown in Table 2. Of the 116 pharmacists who responded to this part of the questionnaire, most reported creating a complete and updated medication list (*n* = 68, 59%) and identifying medication-related issues (*n* = 92, 79%) at least once a week. Overall, 34% (*n* = 39) had received specific training on how to perform structured medication reviews, and of the 38 respondents with a FPH in community pharmacy, 76% (*n* = 29) had received specific training on medication reviews. 98 (85%) pharmacists reported encountering a situation in which deprescribing would be possible at least once a week. Pharmacists that reported to conduct medication reviews stated that the medication review process takes an average of 31 min (*SD* = 32).

**Table 2:** Characteristics of participating pharmacists (*n* = 138)

Gender ^a^	
Female, *n (%)*	113 (82%)
Male, *n (%)*	25 (18%)
**Age in years**	
*Mean (SD)*	44 (11)
Missing, *n (%)*	3 (2%)
**Working settings** (multiple responses possible)	
Community pharmacy, *n (%)*	121 (88%)
Community pharmacy combined with drugstore, *n (%)*	11 (8%)
Hospital,* n (%)*	12 (9%)
Homecare, *n (%)*	1 (1%)
Nursing home *n (%)*	4 (3%)
**Do you work in a place where self-dispensing by physicians is permitted?** ^b^	
Yes *n (%)*	50 (36%)
No *n (%)*	70 (51%)
Mixed system *n (%)*	16 (12%)
Missing *n (%)*	2 (2%)
**Do you have one of the following further training certifications?** (multiple responses possible)	
FPH certificate in anamneses and primary care *n (%)* ^c^	57 (41%)
FPH certificate in community pharmacy *n (%)* ^c^	43 (31%)
FPH certificate in vaccination and blood sampling	94 (68%)
FPH certificate in pharmaceutical counselling for healthcare institutions	3 (4%)
Further training (other FPH certificates/titles) *n (%)* ^c^	24 (17%)
Certificate of Advanced Studies (CAS)/Master of Advanced Studies (MAS) *n (%)*	16 (12%)
PhD *n (%)*	13 (9%)
**How many years have you been working as a pharmacist?**	
*Mean (SD)*	18 (11)
Missing *n (%)*	16 (12%)
**Estimate the percentage of daily interactions with patients ≥ 70 years old with polypharmacy**	
*Mean (SD)*	40 (22)
Missing *n (%)*	17 (12%)

Variables for which missingness was not reported, had no missing responses

^a^None of the participants chose the responses ‘non-binary’ or ‘Do not want to report’ for this question

^b^In Switzerland, self-dispensing cantons are regions in which physicians can dispense medications directly to their patients. In non-self-dispensing cantons medication dispensing is restricted to pharmacists. In mixed cantons, the legislation varies within the canton

^c^FPH, * Foederatio Pharmaceutica Helvetiae* is the certification organisation for pharmacists in Switzerland, which oversees postgraduate and continued education

**Fig. 1:**
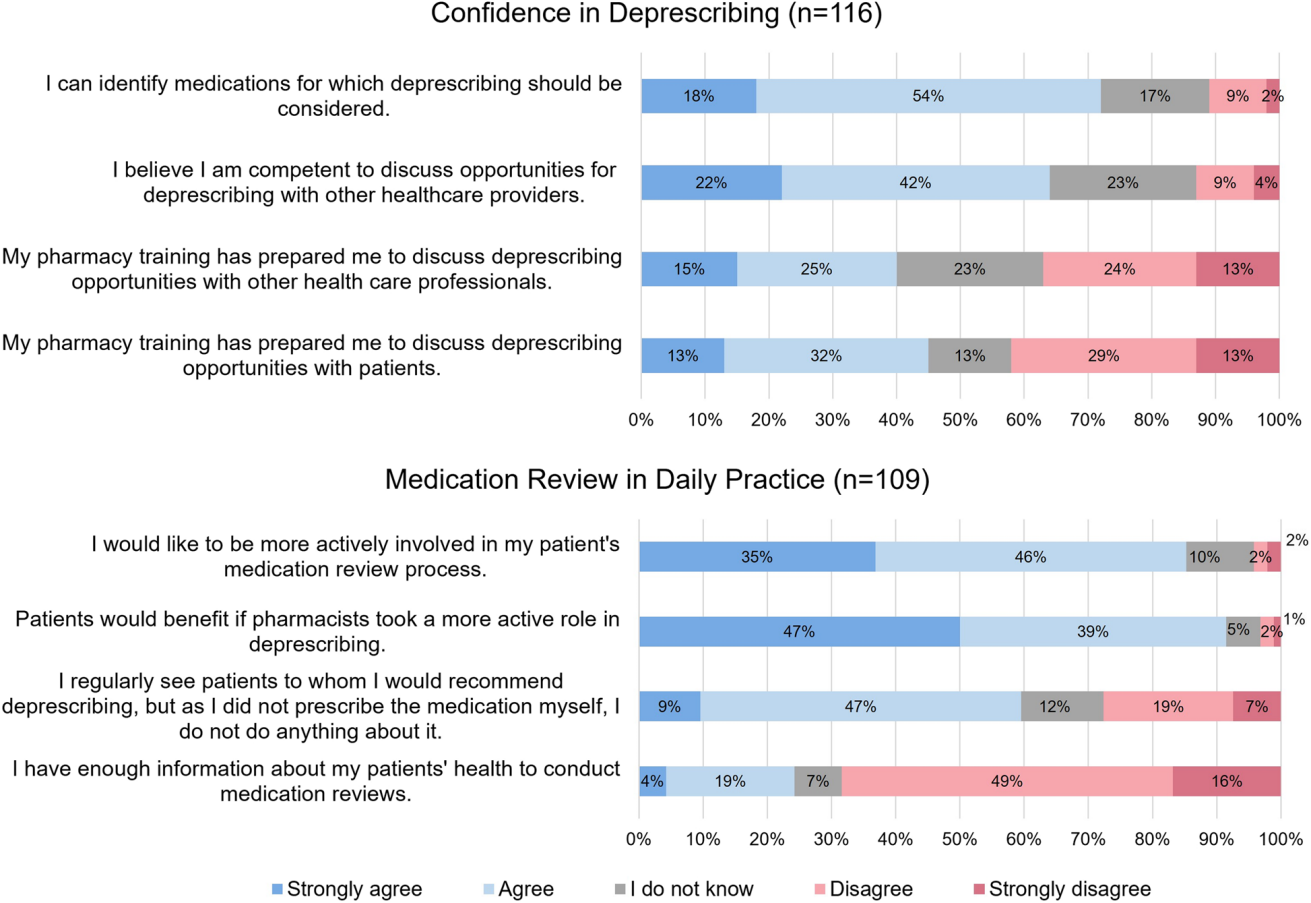
Pharmacists’ views on medication review and their confidence in undertaking deprescribing behaviours. Questions were adapted from Heinrich et al. 2022 [18]

Pharmacists’ confidence in undertaking deprescribing behaviours and medication review in daily practice is shown in Fig. 1 83 (72%) of 116 pharmacists agreed or strongly agreed in being able to identify suitable deprescribing targets, while 49 (42%) disagreed or strongly disagreed that their pharmacy training prepared them to discuss deprescribing opportunities with patients. Regarding medication reviews, 81% (*n* = 88) of the 109 respondents reported that they would like to be more involved in the process of medication reviews, but 65% (*n* = 70) disagreed or strongly disagreed with having enough information about their patients’ health status to conduct medication reviews. 56% (*n* = 61) reported to often see patients for whom they would recommend deprescribing, but as they were not the prescriber they do not react in this situation (e.g., contacting their physician).

### Case vignettes

All pharmacists were willing to deprescribe at least one medication in each case vignette. Pharmacists suggested an average of 4 (*SD* = 3) medications for deprescribing in case vignette 1 (low dependency in ADL), and an average of 3 (*SD* = 3) medications for vignettes 2 and 3 (medium and high dependency in ADL). When comparing deprescribing recommendations by case vignette, pharmacists were less willing to deprescribe for patients with a history of CVD in all case vignettes (Additional file 3). For instance, in case vignette 1 (low dependency in ADL), the difference of willingness to deprescribe at least one medication between patients without and with history of CVD was 24% (95%CI 12% to 36%). This difference of willingness to deprescribe for patients with and without CVD decreased to 10% (2% to 23%) for patients with higher dependency in ADL. In addition, the percentages of medications suggested for deprescribing tended to be lower with higher level of dependency in ADL (Additional file 3). When exploring the willingness to deprescribe by medication type (Additional file 4), we found that the willingness to deprescribe was lower for all medications in all case vignettes when the patient had a history of cardiovascular disease. For instance, in case vignette 1 (low dependency in ADL), pharmacists’ willingness to deprescribe aspirin fell from 41 to 1% when the same hypothetical patient was presented with a history of cardiovascular disease, and for pantoprazole from 65 to 47%. The history of CVD had a lower impact on the willingness to deprescribe antihypertensive medications (e.g., enalapril; decrease from 6 to 3%), for which pharmacists’ willingness to deprescribe was low to begin with. Of the 98 pharmacists who responded to the case vignetter-related questions, 89% (*n* = 87) responded that they perceived enalapril as the most important medication for the patient in case vignette 1 (low cardiovascular risk and low level of dependency in ADL), and 86% (*n* = 84) that pantoprazole as the least important. In all case vignettes, the most common reason reported for deprescribing was the possibility of adverse events (case vignette 1: *n* = 68, 69%; case vignette 2: *n* = 71, 72%; case vignette 3: *n* = 64, 65%).

### Association of patient and pharmacist characteristics with pharmacists’ willingness to deprescribe

The associations between pharmacists’ willingness to make deprescribing recommendations and patients’ history of CVD and level of dependency in ADL are shown in Table 3. The odds of recommending deprescribing were lower in patients with a history of CVD (OR = 0.27, 95%CI 0.21 to 0.36) and lower in patients with higher dependency in ADL compared with low dependency (medium dependency: OR = 0.68, 95%CI 0.54 to 0.87, high dependency: OR = 0.72, 95%CI 0.56 to 0.91). However, the joint presence of medium/high dependency in activities of daily living and a history of CVD increased the odds of making a deprescribing suggestion (CVD x medium dependency: OR = 1.61 95%CI 1.11 to 2.33, CVD x high dependency: OR = 1.75 95%CI 1.21 to 2.52). In sensitivity analysis cases with higher levels of dependency in ADL were at lower odds of willingness to recommend deprescribing only in cases without history of CVD (medium versus low dependency: OR = 0.69, 95%CI 0.54 to 0.87, high versus low dependency: OR = 0.72, 95%CI 0.57 to 0.91), but it was different in cases with history of CVD (medium versus low dependency: OR = 1.10, 95%CI 0.83 to 1.47, high versus low dependency: OR = 1.26, 95%CI 0.95 to 1.67) (Additional file 5). The odds of recommending deprescribing were also higher for pharmacists that had received a training in medication review (OR = 2.48, 95%CI 1.38 to 4.44).

**Table 3:** Current practices of pharmacists working in Switzerland related to conducting medication reviews and attitudes towards deprescribing (*n* = 116)^#^

**Question**	n (%)
**Medication optimisation**	
**I ask patients questions to assess adherence to medication therapy** ^a^	
At least once a week	101 (87%)
Less often than once a week	14 (12%)
Missing	1 (1%)
**I review all medications (prescription, over-the-counter medications, herbals, and supplements) with the patient to create an updated and complete medication list** ^a^	
At least once a week	68 (59%)
Less often than once a week	47 (40%)
Missing	1 (1%)
**I review complete medication list to identify medication-related issues** ^a^	
At least once a week	92 (79%)
Less often than once a week	24 (21%)
**How long does the medication review process take for you?**	
Minutes, mean (SD)	31 (32)
**Which tools do you use to check medication appropriateness of patients ≥ 70 years with > 5 medications? (multiple responses possible)** ^b^	
Lists of potentially inappropriate medications (e.g., Priscus, Beers, START/STOPP)	42 (36%)
Documents/tools for polymedication check ^c^	46 (40%)
Other interaction databases (e.g. Pharmavista, Compendium)	95 (82%)
Other	13 (11%)
**Have you ever received training on how to conduct a detailed medication reviews?**	
Yes (versus no)	39 (34%)
**If yes, did this training take place during your studies at university or afterwards?** ^b^	
At university	13 (33%)
In further education/training	21 (54%)
Other	5 (13%)
**Attitudes towards deprescribing**	
**From 1 to 10, how familiar were you with deprescribing before starting this questionnaire?** ^d^	
Low familiarity (1–3)	32 (28%)
Average familiarity (4–7)	54 (47%)
High familiarity (8–10)	30 (26%)
**What priority should deprescribing have in your daily work?**	
High/very high priority	58 (50%)
Neither high nor low priority/undecided	48 (41%)
No priority/low priority	10 (9%)
**How often does a situation arise in your daily work in which deprescribing would be possible?** ^b^	
Everyday	24 (21%)
Several times a week	42 (36%)
Once a week	32 (28%)
Once a month	11 (10%)
Fewer than that	7 (6%)

^#^Of the 138 pharmacists, 22 (16%) stopped responding the questionnaire in this section. Therefore, the percentages are regarding the total of 116 who responded to this session to the questionnaire

^a^Adapted from Bradley et al. 2018 [41]

^b^No one responded “none”/ “never” to these questions

^c^*Polymedication* check, Medication review tool used in Swiss pharmacies with patients taking ≥ 4 medications for longer than 3 months [34]

^d^Score varying from 0 to 10. Higher scores indicate higher familiarity with the concept of deprescribing

### Interprofessional collaboration in the context of medication review and deprescribing

Pharmacists’ experiences with interprofessional collaboration between pharmacists and physicians with regards to deprescribing are reported in Tables 4 and 5. 97% (*n* = 105) of the pharmacists reported to interact with physicians to clarify questions regarding prescriptions at least once a week. 65% (*n* = 68) of respondents stated that they believe their communication with physicians to be two-way, and 59% (*n* = 64) reported having an interest in supporting physicians to improve their prescribing practices (Additional file 6). Additional file 7 presents pharmacists’ ideas to improve collaboration between pharmacists and general practitioners with regards to medication optimisation based on the free text responses provided: respondents wished for more shared decision-making (*n* = 32, 43%), more efficient ways to communicate with physicians (*n* = 31, 40%), and acceptance of their recommendations and expertise by physicians (*n* = 25, 33%).

**Table 4:** Association between making deprescribing recommendations in each case vignette and the patients’ history of cardiovascular disease and dependency in activities in daily living (ADL), and pharmacists’ characteristics (*n* = 98 pharmacists, *n* = 4,788 observations)

	Crude Odds Ratio (95% CI)	*p*-value	Adjusted Odds Ratio (95% CI)^a^	*p*-value^a^
**Cardiovascular disease (CVD)** (ref: No history of cardiovascular disease)				
History of cardiovascular disease	0.39 (0.33 to 0.45)	0.000	0.27 (0.21 to 0.36)	0.000
**Dependency in activities of daily living (ADL)* (ref: Low)***				
Medium	0.84 (0.70 to 1.00)	0.052	0.68 (0.54 to 0.87)	0.002
High	0.91 (0.76 to 1.08)	0.281	0.72 (0.56 to 0.91)	0.006
**Interaction Terms** *(ref:* CVD x low dependency*)*				
CVD x medium dependency			1.61 (1.11 to 2.33)	0.012
CVD x high dependency			1.75 (1.21 to 2.52)	0.003
**Pharmacist age**				
*Per 10-year increase*	0.86 (0.66 to 1.12)	0.262	0.93 (0.77 to 1.14)	0.629
**Gender** *(*ref: male)				
Female	0.81 (0.39 to 1.70)	0.577	0.77 (0.38 to 1.56)	0.465
**Frequency of seeing patients ≥ 70 years old with polypharmacy (0–100)**				
*Per 10-percentage increase*	0.90 (0.80 to 1.02)	0.110	0.88 (0.78 to 0.99)	0.041
**FPH in community pharmacy** *(*ref: not having a FPH title in community pharmacy)				
Specialized in community pharmacy	0.78 (0.58 to 1.34)	0.406	0.84 (0.47 to 1.52)	0.573
**Training in Medication Review** (ref. not having a training in medication review)				
Having a medication review training	2.42 (1.38 to 4.26)	0.002	2.48 (1.38 to 4.44)	0.002

*FPH Foederatio Pharmaceutica Helvetiae* is the certification organisation for pharmacists in Switzerland, which oversees postgraduate and continued education. The FPH in community pharmacy is required in order to obtain authorization to practice as a pharmacist in the private sector under their own professional responsibility and to bill the compulsory health insurance

^a^Multilevel logistic regression adjusted for patients’ and pharmacists’ characteristics. Dependent variable: Willing to deprescribe each medication. ICC: 0.351

**Table 5:** Pharmacists’ experiences with interprofessional collaboration between pharmacists and physicians with regards to deprescribing (*n* = 109^¥^)

Question	Mean (SD) or n (%)
***How often do you interact with physicians to clarify questions regarding medications prescribed to your patients?***	
Everyday	43 (40%)
Several times a week	46 (42%)
Once a week	16 (15%)
Once a month	2 (2%)
Rarely	2 (2%)
Never	0 (0%)
**How often do you make suggestions to physicians about patients’ medication use?**	
Everyday	19 (18%)
Several times a week	30 (28%)
Once a week	28 (26%)
Once a month	16 (15%)
Rarely	15 (14%)
Never	0 (0%)
**Score of the interprofessional collaboration with physicians (min. 10 to max. 70)**^#^	
*Mean (SD)*	45 (10)

*SD* Standard Deviation

^¥^Missing: 29 (21%) stopped responding the questionnaire in this section. Percentages are regarding the 109 pharmacists who responded to this section

^#^Score range, 10 – 70, adapted from Zillich et al. 2006 [42]. Higher scores indicate greater collaboration

## Discussion

In our sample of pharmacists working mainly in community pharmacies in Switzerland, all were willing to deprescribe at least one medication in each case vignette of oldest-old adults with polypharmacy. The willingness to recommend deprescribing was lower in patients with a history of CVD and lower in patients with higher dependency in ADL. However, the joint presence of medium/high dependency in activities of daily living and a history of CVD increased the odds of making a deprescribing suggestion. Pharmacists who reported having a specific training on structured medication review were more likely to recommend deprescribing. Most pharmacists perceived themselves as capable of identifying drugs suitable for deprescribing and were willing to be more involved in the process of optimising medication use. Regarding their collaboration with physicians in medication reviews, pharmacists wished for more shared decision-making, and more efficient ways to communicate with physicians.

Only a third of pharmacists reported having had sufficient training on how to conduct structured medication reviews and deprescribing, which highlights the need for expanded training opportunities in this area. Of note, it must be considered that only a minority of the participants had a specific training (FPH) in community pharmacy and that their mean age was 41 years, which means that they are likely not representative of the most recent generation of pharmacy graduates in Switzerland. Since 2018, it has been mandatory for all pharmacists to obtain the federal postgraduate title FPH in community pharmacy to obtain a licence allowing them to practice under their own professional responsibility and to bill health insurances [45]. This FPH in community pharmacy includes training on medication reviews. Had our study focused on recent graduates, our results may have been different.

Pharmacists reported that on average 40% of their daily patients are ≥ 70 years old and have polypharmacy. These daily interactions could be a great opportunity to identify and manage situations of inappropriate polypharmacy, which are common among older adults [46]. Most of the pharmacists reported reviewing patients’ medication lists at least once a week, and to take on average half an hour to perform medication reviews. However, previous studies have shown that performing medication reviews can take up to 2–3 h [47, 48]. This discrepancy may be explained by the facts that the term *medication review* can be interpreted in different ways, as there is no universally accepted definition for this service [40]. As most pharmacists in our sample reported not being trained on this service, highlights the need for further education.

Even though only a third of our sample reported having been trained on performing medication reviews, most participants reported being confident in identifying deprescribing opportunities and discussing them with other healthcare providers. These findings could reflect different aspects: On the one hand, this could indicate that pharmacists in our study felt confident in analysing medication lists despite not having received specialised training. On the other hand, this finding could also indicate an overconfidence in the ability to assess medication lists, which could also be reflected in the lower amount of time spent on medication reviews. Finally, pharmacists in our sample could have had different views on what a medication review is. as the definition of medication review varies and can be interpreted differently. For instance, in Switzerland pharmacists provide different services in which they are asked to check medication lists (e.g., “polymedication check” [34]), but those are not exactly a structured medication review.

Most of the pharmacists reported being confident in implementing deprescribing, in line with a study in Ireland [18]. Nevertheless, in our study, less than half reported being confident in discussing deprescribing suggestions with patients, which highlights the need of training on patient involvement in medication optimisation. Furthermore, the finding that more than half of the respondents reported to not react to deprescribing opportunities (e.g., not contact physician with specific suggestions) could indicate a lack of “ownership” of what medications they dispense to their patients. This is plausible in the context of the findings from another study, which found pharmacists to be hesitant to make deprescribing recommendations to physicians [30]. Nevertheless, pharmacists are legally equally responsible for the medications dispensed as the prescribing physicians. A further explanation for this inertia could be the lack of knowledge about their patients’ health history, since most pharmacists reported lacking information on this aspect. More access to complete patient health records has indeed been shown to allow pharmacists to make better-informed deprescribing recommendations based on patients’ health status, and to share these recommendations more efficiently [49].

Pharmacists in our study were willing to make deprescribing recommendations for patients aged ≥ 80 years with polypharmacy, and all were willing to deprescribe at least one medication in each case vignette. In the LESS study with general practitioners [43, 50], in which the same case vignettes were used in 31 countries, GPs’ willingness to deprescribe was lower compared to the pharmacists’ willingness in the present study. Both our study and those with GPs found the willingness to deprescribe to be lower in patients with a history of CVD. In our study, the odds of recommending deprescribing were lower for patients with higher dependency in ADL. However, as the effect of dependency in ADL on the outcome was significantly modified by the history of CVD, this finding should be interpreted with caution. When considering only patients without a history of CVD, pharmacists’ willingness to deprescribe was lower in patients with higher dependency in ADL, but it was not the case for patients with history of CVD. Interestingly, previous studies with GPs reported the willingness to deprescribe to be higher with higher dependency in ADL [43, 50, 51]. In addition, pharmacists who reported having received a specific training on how to perform structured medication reviews were more willing to deprescribe, which highlights again the importance of education.

The history of CVD seemed to have a greater impact on pharmacists’ deprescribing choices than in GPs’ choices, especially regarding cardiovascular medications [43]. For instance, in this present study, in case vignette 1 (low dependency in ADL) pharmacists’ willingness to deprescribe aspirin fell from 41 to 1% and pantoprazole from 65 to 47% once the hypothetical patient was presented with a history of cardiovascular disease. For antihypertensive medications, the history of CVD had a low impact on the willingness to deprescribe, which is in line with the GP study using the same case vignettes [43]. Proton pump inhibitors were the medication most commonly chosen for deprescribing in all cases vignettes, in line with the GP study [43]. However, when Swiss GPs received the same case vignettes [50], cardiovascular preventive medications like atorvastatin were the most commonly chosen deprescribing candidate, and pantoprazole was the second.

In our study, the most commonly reported reason for deprescribing was the risk of adverse events, followed by lack of benefits, which is in line with the study with GPs [43]. The similarities in the deprescribing decisions of pharmacists in our study and GPs who responded to the same case vignettes evidence the feasibility of collaboration between these professionals in the context of deprescribing. Other studies have reported that physicians are willing to accept deprescribing recommendations from pharmacists, and their similar decisions could be an enabler for their collaboration [29, 30]. We also identified several barriers to the collaboration between pharmacists and physicians in the context of medication optimisation. Pharmacists in our study wished for more opportunities to interact with physicians, quicker and more efficient communication channels between them, more opportunities for shared decision-making between them, and more access to patient information, which is in line with other studies [31, 52–54].

Our findings have significant implications for clinical practice and future research on medication optimisation within the context of Swiss primary care settings. The high willingness of pharmacists to make deprescribing recommendations, their confidence in identifying deprescribing opportunities, and their wish to be more involved in this process indicate that the involvement of pharmacists can facilitate the implementation of deprescribing and medication optimisation efforts. In addition, our study highlights the need of more training on medication review offered to pharmacists, including information on deprescribing-related communication with patients and physicians. Our study raises awareness of the need to facilitate interprofessional collaboration between physicians and pharmacists in the context of medication optimisation. To improve the implementation of medication reviews, future interventions should focus on ways to improve communication between pharmacists and physicians, shared decision-making between them, and access to patient information.

Our survey study is strengthened by the fact that we invited a random sample of Swiss pharmacists to participate in our study. Indeed, pharmacists working in 20 out of 26 cantons in the different language regions of Switzerland completed the survey. Nevertheless, our findings may not be generalizable to other countries. Our study also comes with several limitations. First, the use of hypothetical case vignettes may not fully capture how pharmacists regularly manage older adults with polypharmacy in real-life clinical practice. Similarly, the self-reported information on pharmacist-physician collaboration may not reflect real-world collaboration. Second, we cannot rule out volunteer bias, as the pharmacists who participated in our study may be more interested in medication optimisation than those who chose not to participate. Third, we managed to recruit the target sample size, but the fact that not all pharmacists responded to all the questions decreased our sample size for some analyses. For feasibility reasons, we were unable to extend the recruitment period. The regression model however was performed at the medication level for the case vignettes, which allowed for a sufficiently big sample. Finally, we did not collect specific reasons for deprescribing by medication type, which is why we are unable to compare across medication types.

## Conclusion

All pharmacists in this study were willing to recommend deprescribing for at least one medication in oldest-old patients with polypharmacy. Willingness was higher for patients with lower cardiovascular risk and lower in patients with higher dependency in ADL. Pharmacists were confident in their capacity to make deprescribing recommendations and would like to be more involved in the process of medication review and deprescribing, which provides great potential for medication optimisation efforts in Swiss primary care settings.

### Supplementary Information

Additional file 1: Figure S1. Workplace locations of respondents

Additional file 2: Table S1. Pharmacists’ deprescribing recommendations per case vignette.

Additional file 3: Table S2. Percentages of pharmacists' willingness to deprescribe each medication by case vignette according to the medication type, history of cardiovascular disease, and dependency level.

Additional file 4: Table S3. Sensitivity analysis of the association between making deprescribing recommendations in each case vignette, dependency in activities in daily living and pharmacists’ characteristics by patients’ history of cardiovascular disease.

Additional file 5: Figure S2. Interprofessional collaboration between pharmacists and physicians.

Additional file 6: Table S4. Suggestions made by pharmacists on how to improve collaboration between pharmacists and general practitioners with regards to medication optimisation in Swiss primary care settings.

Additional file 7. Study questionnaire for pharmacists.

## Data Availability

The dataset used and analysed during the current study is available from the corresponding author upon reasonable request. The questionnaire used in this study is available in the Additional file 1.
