# Supplementary material for: Pharmacists’ attitudes towards interprofessional collaboration to optimise medication use in older patients in Switzerland: a survey study
Source: BMC Health Serv Res. 2024 Jul 26;24:849. doi: 10.1186/s12913-024-11339-8 (PMC11282592; doi:10.1186/s12913-024-11339-8)
Supplement: Supplementary file 2 — Additional file 2: Table S1. Pharmacists’ deprescribing recommendations per case vignette. [file 12913_2024_11339_MOESM2_ESM.docx]

## **Pharmacists’ attitudes towards interprofessional collaboration to optimise medication use in older patients in Switzerland: A survey study**

Renata Vidonscky Lüthold^1,2^, Damien Cateau^3^, Stephen Philip Jenkinson^1,3^, Sven Streit^1,a^, Katharina Tabea Jungo^1,4,a^

^1^Institute of Primary Health Care (BIHAM), University of Bern, 3012 Bern, Switzerland.

^2^Graduate School for Health Sciences, University of Bern, Bern, Switzerland.

^3^Centre for Primary Care and Public Health (Unisanté), University of Lausanne, Lausanne, Switzerland.

^4^Division of Pharmacoepidemiology and Pharmacoeconomics and Center for Healthcare Delivery Sciences (C4HDS), Department of Medicine, Brigham and Women's Hospital and Harvard Medical School, 02115 Boston, MA, United States of America

^a^ SS and KTJ share last co-authorship

**Additional File 3 - Table s1.** Pharmacists’ deprescribing recommendations per case vignette (n=138)^#^

| Case vignette | Patients’ dependency level | Deprescribing recommendation | No history of CVD^a^  (95%CI) | With history of CVD^a^  (95%CI) | Difference  (95% CI)^b^ |
| --- | --- | --- | --- | --- | --- |
| 1 | low | Min. 1 medication | 79% (72% to 87%) | 55% (46% to 64%) | 24% (12% to 36%) |
|  |  | Min. 2 medication | 75% (67% to 83%) | 36% (27% to 45%) | 39% (27% to 51%) |
|  |  | Min. 3 medication | 53% (43% to 62%) | 16% (10% to 23%) | 36% (25% to 48%) |
| 2 | medium | Min. 1 medication | 69% (61% to 77%) | 57% (48% to 66%) | 12% (-1% to 25%) |
|  |  | Min. 2 medication | 60% (51% to 69%) | 42% (33% to 51%) | 18% (5% to 31%) |
|  |  | Min. 3 medication | 41% (32% to 49%) | 17% (10% to 24%) | 23% (12% to 35%) |
| 3 | high | Min. 1 medication | 66% (57% to 74%) | 55% (46% to 64%) | 10% (2% to 23%) |
|  |  | Min. 2 medication | 58% (49% to 67%) | 44% (35% to 53%) | 14% (1% to 27%) |
|  |  | Min. 3 medication | 41% (32% to 50%) | 20% (13% to 27%) | 22% (10% to 33%) |

Missing = 40.

CI: Confidence interval

^a^CVD= Cardiovascular disease

^b^ Two-sample test of proportions
