# Supplementary material for: Pharmacists’ attitudes towards interprofessional collaboration to optimise medication use in older patients in Switzerland: a survey study
Source: BMC Health Serv Res. 2024 Jul 26;24:849. doi: 10.1186/s12913-024-11339-8 (PMC11282592; doi:10.1186/s12913-024-11339-8)
Supplement: Supplementary file 3 — Additional file 3: Table S2. Percentages of pharmacists' willingness to deprescribe each medication by case vignette according to the medication type, history of cardiovascular disease, and dependency level. [file 12913_2024_11339_MOESM3_ESM.docx]

## **Pharmacists’ attitudes towards interprofessional collaboration to optimise medication use in older patients in Switzerland: A survey study**

Renata Vidonscky Lüthold^1,2^, Damien Cateau^3^, Stephen Philip Jenkinson^1,3^, Sven Streit^1,a^, Katharina Tabea Jungo^1,4,a^

^1^Institute of Primary Health Care (BIHAM), University of Bern, 3012 Bern, Switzerland.

^2^Graduate School for Health Sciences, University of Bern, Bern, Switzerland.

^3^Centre for Primary Care and Public Health (Unisanté), University of Lausanne, Lausanne, Switzerland.

^4^Division of Pharmacoepidemiology and Pharmacoeconomics and Center for Healthcare Delivery Sciences (C4HDS), Department of Medicine, Brigham and Women's Hospital and Harvard Medical School, 02115 Boston, MA, United States of America

^a^ SS and KTJ share last co-authorship

**Additional File 4 - Table s2.** Percentages of pharmacists' willingness to deprescribe each medication by case vignette according to the medication type, history of cardiovascular disease, and dependency level (n= 116)

| Medication | Level of dependency in activities of daily living | | |
| --- | --- | --- | --- |
|  | Low  (Case vignette 1) | Medium  (Case vignette 2) | High  (Case vignette 3) |
|  | Percentage of Pharmacists (95% CI) | Percentage of Pharmacists (95% CI) | Percentage of Pharmacists (95% CI) |
| *Pain medications* | | | |
| **Tramadol 50 mg, twice daily** | | | |
| Without history of CVD | 50% (41% to 59%) | 54% (45% to 64%) | 51% (41% to 60%) |
| With history of CVD | 33% (24% to 42%) | 46% (36% to 55%) | 44% (35% to 53%) |
| **Paracetamol 1 g, three times daily** | | | |
| Without history of CVD | 27% (13% to 36%) | 19% (12% to 27%) | 19% (12% to 27%) |
| With history of CVD | 16% (10% to 24%) | 15% (9% to 22%) | 16% (9% to 23%) |
| *Proton-pump inhibitor* | | | |
| **Pantoprazole 20 mg, once daily** | | | |
| Without history of CVD | 65% (55% to 73%) | 56% (47% to 65%) | 53% (43% to 62%) |
| With history of CVD | 47% (38% to 57%) | 46% (36% to 55%) | 44% (35% to 53%) |
| *Antihypertensive medications* | | | |
| **Amlodipine 5 mg, once daily** | | | |
| Without history of CVD | 15% (9% to 22%) | 14% (8% to 21%) | 15% (9% to 22%) |
| With history of CVD | 6% (2% to 12%) | 4% (1% to 10%) | 8% (4% to 14%) |
| **Enalapril 10 mg, once daily** | | | |
| Without history of CVD | 6% (2% to 12%) | 4% (1% to 10%) | 8% (4% to 14%) |
| With history of CVD | 3% (1% to 7%) | 3% (1% to 7%) | 4% (1% to 10%) |
| *Cholesterol-lowering medication* | | | |
| **Atorvastatin 40 mg, once daily** | | | |
| Without history of CVD | 42% (33% to 52%) | 26% (18% to 35%) | 36% (27% to 46%) |
| With history of CVD | 5% (2% to 11%) | 5% (2% to 11%) | 12% (7% to 19%) |
| *Antiplatelet medication* | | | |
| **Aspirin 100 mg, once daily** | | | |
| Without history of CVD | 41% (32% to 51%) | 28% (20% to 37%) | 25% (17% to 34%) |
| With history of CVD | 1% (0% to 5%) | 1% (0% to 5%) | 3% (1% to 7%) |

CI: Confidence interval; CVD: Cardiovascular disease; GP: General practitioner

Missing: Of the 138 respondents, there were 40 missing.
