# Supplementary material for: Pharmacists’ attitudes towards interprofessional collaboration to optimise medication use in older patients in Switzerland: a survey study
Source: BMC Health Serv Res. 2024 Jul 26;24:849. doi: 10.1186/s12913-024-11339-8 (PMC11282592; doi:10.1186/s12913-024-11339-8)
Supplement: Supplementary file 4 — Additional file 4: Table S3. Sensitivity analysis of the association between making deprescribing recommendations in each case vignette, dependency in activities in daily living and pharmacists’ characteristics by patients’ history of cardiovascular disease. [file 12913_2024_11339_MOESM4_ESM.docx]

## **Pharmacists’ attitudes towards interprofessional collaboration to optimise medication use in older patients in Switzerland: A survey study**

Renata Vidonscky Lüthold^1,2^, Damien Cateau^3^, Stephen Philip Jenkinson^1,3^, Sven Streit^1,a^, Katharina Tabea Jungo^1,4,a^

^1^Institute of Primary Health Care (BIHAM), University of Bern, 3012 Bern, Switzerland.

^2^Graduate School for Health Sciences, University of Bern, Bern, Switzerland.

^3^Centre for Primary Care and Public Health (Unisanté), University of Lausanne, Lausanne, Switzerland.

^4^Division of Pharmacoepidemiology and Pharmacoeconomics and Center for Healthcare Delivery Sciences (C4HDS), Department of Medicine, Brigham and Women's Hospital and Harvard Medical School, 02115 Boston, MA, United States of America

^a^ SS and KTJ share last co-authorship

**Additional File 5 - Table s3.** Sensitivity analysis of the association between making deprescribing recommendations in each case vignette, dependency in activities in daily living and pharmacists’ characteristics by patients’ history of cardiovascular disease (n=98 pharmacists, n=2,394 observations)

|  | Adjusted Odds Ratio (95% CI) ^a^ | p-value ^a^ |
| --- | --- | --- |
| **Patients without cardiovascular disease** | | |
| **Dependency in activities of daily living (ADL)** *(ref: low)* | | |
| Medium | 0.69 (0.54 to 0.87) | 0.002 |
| High | 0.72 (0.57 to 0.91) | 0.006 |
| **Pharmacist age** | | |
| *Per 10-year increase* | 0.98 (0.76 to 1.26) | 0.881 |
| **Gender** *(ref: male)* | | |
| Female | 0.89 (0.46 to 1.71) | 0.717 |
| **Frequency of seeing patients ≥70 years old with polypharmacy (0-100)** | | |
| *Per 10-percentage increase* | 0.88 (0.79 to 0.99) | 0.026 |
| **FPH in community pharmacy** *(ref: not having a FPH title in community pharmacy)* | | |
| Specialized in community pharmacy | 0.88 (0.52 to 1.51) | 0.651 |
| **Training in Medication Review** *(ref. not having a training in medication review)* | | |
| Having a medication review training | 2.37 (1.39 to 4.06) | 0.002 |
| **Patients with cardiovascular disease** | | |
| **Dependency in activities of daily living (ADL)** *(ref: low)* | | |
| Medium | 1.10 (0.83 to 1.47) | 0.510 |
| High | 1.26 (0.95 to 1.67) | 0.113 |
| **Pharmacist age** | | |
| *Per 10-year increase* | 0.87 (0.65 to 1.17) | 0.360 |
| **Gender** *(ref: male)* | | |
| Female | 0.66 (0.31 to 1.42) | 0.290 |
| **Frequency of seeing patients ≥70 years old with polypharmacy (0-100)** | | |
| *Per 10-percentage increase* | 0.91 (0.80 to 1.04) | 0.163 |
| **FPH in community pharmacy** *(ref: not having a FPH title in community pharmacy)* | | |
| Specialized in community pharmacy | 0.83 (0.44 to 1.57) | 0.574 |
| **Training in Medication Review** *(ref. not having a training in medication review)* | | |
| Having a medication review training | 2.38 (1.28 to 4.44) | 0.006 |

^a^ Multilevel logistic regression adjusted for patients’ and pharmacists’ characteristics. Dependent variable: Willing to deprescribe each medication. ICC: 0.347.

FPH*: Foederatio Pharmaceutica Helvetiae* is the certification organisation for pharmacists in Switzerland, overseeing postgraduate and continued education. The FPH in community pharmacy is required in order to obtain authorization to practice as a pharmacist in the private sector under their own professional responsibility and to bill the compulsory health insurance.
