# Supplementary material for: Pharmacists’ attitudes towards interprofessional collaboration to optimise medication use in older patients in Switzerland: a survey study
Source: BMC Health Serv Res. 2024 Jul 26;24:849. doi: 10.1186/s12913-024-11339-8 (PMC11282592; doi:10.1186/s12913-024-11339-8)
Supplement: Supplementary file 5 — Additional file 5: Figure S2. Interprofessional collaboration between pharmacists and physicians. [file 12913_2024_11339_MOESM5_ESM.docx]

## **Pharmacists’ attitudes towards interprofessional collaboration to optimise medication use in older patients in Switzerland: A survey study**

Renata Vidonscky Lüthold^1,2^, Damien Cateau^3^, Stephen Philip Jenkinson^1,3^, Sven Streit^1,a^, Katharina Tabea Jungo^1,4,a^

^1^Institute of Primary Health Care (BIHAM), University of Bern, 3012 Bern, Switzerland.

^2^Graduate School for Health Sciences, University of Bern, Bern, Switzerland.

^3^Centre for Primary Care and Public Health (Unisanté), University of Lausanne, Lausanne, Switzerland.

^4^Division of Pharmacoepidemiology and Pharmacoeconomics and Center for Healthcare Delivery Sciences (C4HDS), Department of Medicine, Brigham and Women's Hospital and Harvard Medical School, 02115 Boston, MA, United States of America

^a^ SS and KTJ share last co-authorship


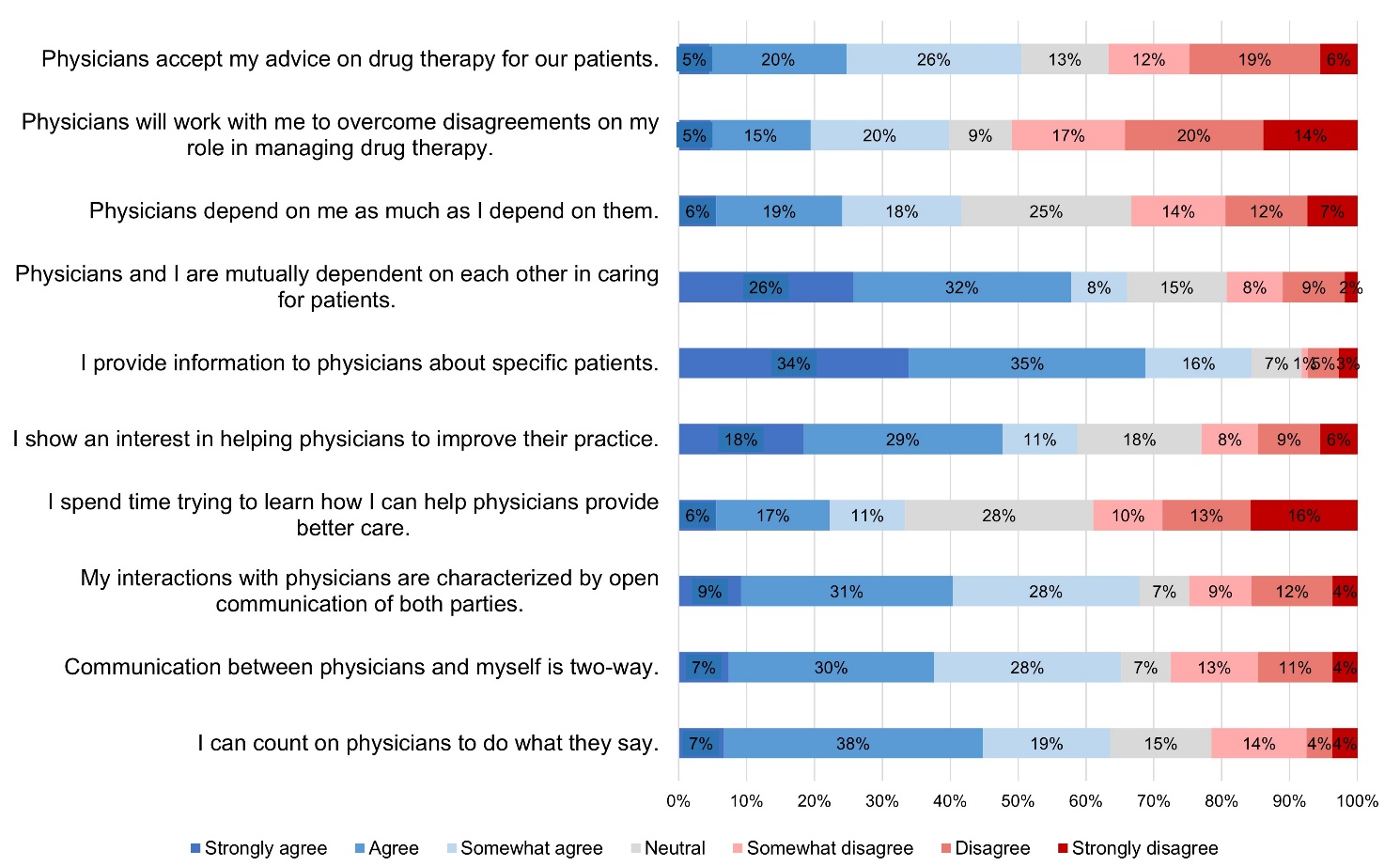


**Additional File 6 – Figure s2.** Interprofessional collaboration between pharmacists and physicians (n=109).

Questions were adapted from Zillich et al., 2006 [1].

References

1. Zillich AJ, Milchak JL, Carter BL, Doucette WR. Utility of a questionnaire to measure physician-pharmacist collaborative relationships. J Am Pharm Assoc (2003). 2006;46(4):453-8.
