# Supplementary material for: Pharmacists’ attitudes towards interprofessional collaboration to optimise medication use in older patients in Switzerland: a survey study
Source: BMC Health Serv Res. 2024 Jul 26;24:849. doi: 10.1186/s12913-024-11339-8 (PMC11282592; doi:10.1186/s12913-024-11339-8)
Supplement: Supplementary file 6 — Additional file 6: Table S4. Suggestions made by pharmacists on how to improve collaboration between pharmacists and general practitioners with regards to medication optimisation in Swiss primary care settings. [file 12913_2024_11339_MOESM6_ESM.docx]

## **Pharmacists’ attitudes towards interprofessional collaboration to optimise medication use in older patients in Switzerland: A survey study**

Renata Vidonscky Lüthold^1,2^, Damien Cateau^3^, Stephen Philip Jenkinson^1,3^, Sven Streit^1,a^, Katharina Tabea Jungo^1,4,a^

^1^Institute of Primary Health Care (BIHAM), University of Bern, 3012 Bern, Switzerland.

^2^Graduate School for Health Sciences, University of Bern, Bern, Switzerland.

^3^Centre for Primary Care and Public Health (Unisanté), University of Lausanne, Lausanne, Switzerland.

^4^Division of Pharmacoepidemiology and Pharmacoeconomics and Center for Healthcare Delivery Sciences (C4HDS), Department of Medicine, Brigham and Women's Hospital and Harvard Medical School, 02115 Boston, MA, United States of America

^a^ SS and KTJ share last co-authorship

**Additional File 7 - Table s4.** Suggestions made by pharmacists on how to improve collaboration between pharmacists and general practitioners with regards to medication optimisation in Swiss primary care settings (n=75)

| **Topic** | **Description** | **n (%)** |
| --- | --- | --- |
| Shared decision-making between pharmacists and physicians | Wish for more interprofessional work practices, teamwork, shared responsibilities, and wish for a higher acceptance of pharmacists’ role by physicians. | 32 (43%) |
| Efficient communication between pharmacists and physicians | Quicker responses from physicians, direct contact between pharmacists and physicians, easier access for pharmacists to physicians, and availability of an electronic platform to facilitate communication and data sharing. | 31 (40%) |
| Equality in the workplace | More acceptance and respect from physicians towards pharmacists’ (de)prescribing recommendations, understanding by physicians that both pharmacists and physician have equal importance in the medication optimisation process. | 25 (33%) |
| Regular meetings between pharmacists and physicians (and other health care professionals) | Regular meetings like *quality circles*^1^, in which physicians, pharmacists and other healthcare professionals discuss questions and issues that arise in their clinical practice. Through regular meetings they could get to know each other better, which would facilitate daily interactions. | 23 (31%) |
| Access to detailed patient health information | Wish from pharmacists for shared digital patient health records that facilitate data sharing and allow them to access complete patients’ health data (pharmacists in Switzerland currently do not have access to diagnoses, complete medication lists including the reasons for prescribing, laboratory or vital data). | 22 (28%) |
| Exchange of physicians’ and pharmacists’ work experiences | Pharmacists visiting physicians’ practices and physicians visiting pharmacies regularly would improve the understanding of both parties on what the daily clinical routines of the other party look like. Wish for more opportunities to interact and discuss patient scenarios. | 19 (25%) |
| Acknowledgement and recognition of the pharmacists’ role and knowledge by physicians | Wish that physicians had more awareness, respect, recognition, and understanding of the pharmacists’ role in patient care. If physicians would learn more about pharmacists’ education, capabilities, knowledge, and skills, this would facilitate interprofessional collaboration. | 17 (23%) |
| Joint training for pharmacists and physicians | Physicians and pharmacists should have joint training sessions/educational opportunities (from the university setting to the continued education setting). Joint events could facilitate the teamwork in daily practice. | 12 (16%) |

Responses to the free-text question “*What would improve collaboration between pharmacists and family doctors?*” were analyzed using a quantitative text analysis.

^1^ Quality circles are regular meetings among healthcare professionals (commonly among general practitioners), in which they discuss their general work practice.
