# Supplementary material for: Pharmacists’ attitudes towards interprofessional collaboration to optimise medication use in older patients in Switzerland: a survey study
Source: BMC Health Serv Res. 2024 Jul 26;24:849. doi: 10.1186/s12913-024-11339-8 (PMC11282592; doi:10.1186/s12913-024-11339-8)
Supplement: Supplementary file 7 — Additional file 7. Study questionnaire for pharmacists. [file 12913_2024_11339_MOESM7_ESM.pdf]

Pharmacists' attitudes towards interprofessional collaboration to optimise medication use in older patients in Switzerland: A survey study

Renata Vidonscky Lüthold[1,2], Damien Cateau[3], Stephen Philip Jenkinson[1,3], Sven Streit[1,a], Katharina Tabea Jungo[1,4,a]

[1]Institute of Primary Health Care (BIHAM), University of Bern, 3012 Bern, Switzerland.
[2]Graduate School for Health Sciences, University of Bern, 3012 Bern, Switzerland.
[3]Centre for Primary Care and Public Health (Unisanté), University of Lausanne, Lausanne, Switzerland.
[4]Division of Pharmacoepidemiology and Pharmacoeconomics and Center for Healthcare Delivery Sciences (C4HDS), Department of Medicine, Brigham and Women's Hospital and Harvard Medical School, 02115 Boston, MA, United States of America

[a] SS and KTJ share last co-authorship

Additional File 1 – Study questionnaire for pharmacists

This document was translated from German to English

Pharmacists' attitudes towards interprofessional collaboration to optimise medication use in older patients in Switzerland

Information for pharmacists

Dear Colleagues,

People on multiple medications (polypharmacy) are at particularly high risk for side effects and overtreatment. While it is often recommended to stop taking inappropriate medications or reduce their dosage (called deprescribing), this approach is challenging for both patients and general practitioners for a variety of reasons. In this context, the involvement of pharmacists and their collaboration with general practitioners can improve the deprescribing process. In this survey, we would like to learn more about pharmacists' views on the process of discontinuing or reducing medications and interprofessional collaboration in this context.

We would like to invite you to take part in this anonymous survey of around 15 - 20 minutes.
This survey contains questions about your working practice, interprofessional collaboration, and your attitude towards reducing or stopping medications.
Do you work in a community pharmacy, hospital, nursing home or homecare facility? Then we cordially invite you to take part in this survey. The data is collected using SurveyMonkey and stored securely on a server at the University of Bern. Your answers will be treated confidentially and the data will be collected anonymously.
Thank you very much for your participation. We - a very motivated interprofessional team - really appreciate that you take the time to participate in this study.

Part A

Background information

For this survey we are interested in the views of pharmacists on the process of stopping potentially harmful medications or reducing their dosage in primary care. The survey was designed for who work in contact with patients.

1. Where do you work (multiple answer options)?
   - o Community pharmacy
   - o Combined pharmacy with drug-store
   - o Hospital
   - o Homecare
   - o Nursing home
   - o Other:(direct branching logic to the end of survey, to the explanation that they are not eligible to participate but still thank them!)

2. Please indicate your age in years: _____

3. Please indicate your gender.

   - o Male
   - o Female
   - o Other (which):
   - o Prefer to not answer.

4. In which canton do you currently work?

o Appenzell Innerrhoden (AI)	o Graubünden (GR)	o Solothurn (SO)
o Appenzell Ausserrhoden (AR)	o Jura (JU)	o Thurgau (TG)
o Aargau (AG)	o Luzern (LU)	o Tessin (TI)
o Bern (BE)	o Neuenburg (NE)	o Uri (UR)
o Basel Stadt (BS)	o Obwalden (OW)	o Wallis (VS)
o Basel Land (BL)	o Nidwalden (NW)	o Waadt (VD)
o Freiburg (FR)	o St. Gallen (SG)	o Zug (ZG)
o Genf (GE)	o Schaffhausen (SH)	o Zürich (ZH)
o Glarus (GL)	o Schwyz (SZ)	

5. Do you regularly work in more than one place?
   - Yes
   - No

6. (If yes) If you work in multiple pharmacies and the answer to a question varies depending on the pharmacy, please refer to the pharmacy where you work most often.

7. Do you work in a place where *self-dispensation* by doctors is permitted?
   - Yes
   - No
   - Mixed canton

8. (If «mixed canton») Can the nearest doctor give the medication directly to the patient?

9. Do you have any of the following further training courses/titles? Please check all that apply.
   - FPH in Anamnese in primary care
   - FPH in vaccination and blood collection
   - FPH in integrated care models
   - FPH consultant pharmacist for outpatient medication prescription
   - FPH in pharmaceutical support for healthcare institutions
   - FPH in community pharmacy
   - FPH is hospital
   - FPH in clinical pharmacy
   - Certificate of Advanced Studies (CAS) / Master of Advanced Studies (MAS)
   - PhD
   - Other:

10. How many years have you been working as pharmacist? (in number of years)

11. How much do you work according to your work contract? Please give your answer as a percentage between 0 and 100. A full-time position corresponds to 100 percent.

For the next few questions, we will use the word “patient” to refer to a person with whom you may interact during your work and provide pharmaceutical services. Please consider each patient/client/customer - of any gender - to answer these questions.

12. How often do you advise/serve patients who fulfil the following criteria?
    - Alter von ≥ 70 Jahren,
    - ≥ 5 Medikamente

    - Multiple times a day
    - Every day
    - Several times a week
    - Rarer

13. Thinking about all the recipes you see in a workday, what percentage of them are electronic and many of them are paper? (If you work in multiple pharmacies and the answer to a question varies depending on the pharmacy, please refer to the pharmacy where you work most often.) Please provide a number between 0-100% for each option.

    - Paper prescriptions sent by fax or scanned and sent by email
    - Recipes created by recipe software (i.e. not handwritten) and printed on paper
    - Prescriptions that are sent purely electronically

14. Do you use an electronic tool to edit and analyze patients' medication lists?
    - Yes
    - No

15. (Branching logic, if «yes»): What kind of electronic tool do you use in your work?

    - eMediplan
    - Other tools, please specify:

Part B

Familiarity with the concept of deprescribing

Deprescribing is commonly defined as the process of stopping inappropriate medications or reducing the dosage thereof, aiming the reduction of polypharmacy and the improvement of quality of life.

16. How familiar are you with the concept of deprescribing?

o 1 (never heard about it)	o 2
o 3	o 4
o 5	o 6
o 7	o 8
o 9	o 10 (very familiar)

17. For those who have heard of it before (branching logic): Where have you learnt about this topic? (Multiple responses possible)
    - scientific literature/magazines
    - Workshops/Further training (e.g., FPH, CAS)
    - Conferences, courses
    - Other (where?):

18. What priority should “deprescribing” have in your daily work?
    - No priority

- o Low priority
- o Neither low nor high priority
- o High priority
- o Very high priority
- o Undecided

19. How often does a situation arise for a possible deprescribing in your daily work??
    - o every day
    - o several times a week
    - o once a week
    - o once a month
    - o less common
    - o never

<u>Confidence in Undertaking Deprescribing Behaviours</u>

For each of the following questions, please indicate which of the statements best reflects your opinion. *We consider your previous pharmacist training to be all the university training, further education, trainings, etc. that you have completed so far.*

	Strongly disagree	Disagree	Neutral	Agree	Strongly Agree
20. My pharmacist training has prepared me to discuss deprescribing opportunities with patients.					
21. My pharmacist training has prepared me to discuss deprescribing opportunities with other health care professionals.					
22. I believe I am competent to discuss opportunities for deprescribing with other health care professionals.					
23. I can identify medications for which deprescribing should be considered.					

Part C

Work practices

<u>Typical work month</u>

Reflecting on a typical month at your workplace, please indicate, on average, how often you perform the following activities when indicated:

	Every working day	Multiple times a week	Once a week	Once a month	Less frequently than that	Never
24. Reviewed all medications (prescription, over- the-counter, herbals, and supplements) with the patient to create an updated and complete medication list						
25. Asked the patient questions to assess adherence to medication therapy						
26. Reviewed complete medication list to identify medication-related issues (i.e. incorrect dose, drug interaction)						

Medication Review in work practice (branching logic: show if response for 23 or 25: something other than Never)

Below you will find some questions about the *Medication review* process in your work practice. By the term *Medication review* we mean a structured evaluation of a patient's medications. This includes identifying medication-related problems and making concrete suggestions for improvement. The goal of a medication review is to identify, solve and prevent drug-related problems in order to generally optimize drug therapy, reduce drug side effects and improve clinical outcomes.

27. Have you ever received any training on how to conduct detailed medication reviews?
    - Yes
    - No

28. If so, did this training take place during your studies at university or after?
    - At university
    - In further education/training
    - Other: __________

29. Regarding patients who are older than 70 years and have polypharmacy (>5 medications): When checking their medications for appropriateness, which instruments/tools/auxiliary materials/etc. use? Check all that apply.
    - Lists of potentially inappropriate medications (e.g., Priscus, Beers, START/STOP)
    - Documents/tools for polymedication check
    - Other interaction databases (e.g. Pharmavista, Compendium)
    - Other:
    - None

For each of the following questions, please indicate which of the statements best reflects your opinion.

	Strongly disagree	Disagree	I do not know	Agree	Strongly Agree
30. I have enough information about my patient to perform medication reviews.					
31. I regularly see patients to whom I would recommend stopping/reducing medication. But since I didn't prescribe the medication myself, I didn't do anything about it.					
32. Patients would benefit if pharmacists had a more active role in deprescribing.					
33. I would like to be more actively involved in my patients' medication review process.					

34. What information are you missing to carry out medication reviews?

35. If you conduct medication reviews, how long does it take you on average? Please provide the answer in minutes.

36. Who do you contact if you determine that a medication should be reduced or discontinued? Check all that apply.

- To no one/I do not speak to anyone
- Directly to the patient
- To the patient’s GP
- To the prescribing doctor
- If available: homecare
- Others:

Part D

Interprofessional collaboration between pharmacists and physicians

In this part of the survey we are interested in the interprofessional collaboration between pharmacists and physicians in the context of deprescribing and medication review.

36. Please indicate your agreement with the statements below.

	strongly disagree	disagree	somewhat disagree	neutral	somewhat agree	agree	strongly agree
I can count on physicians to do what they say.							
Communication between physicians and myself is two-way.							
My interactions with physicians are characterized by open communication of both parties.							
I spend time trying to learn how I can help physicians provide better care.							
I show an interest in helping physicians improve his/her practice.							
I provide information to physicians about specific patients.							
Physicians and I are mutually dependent on each other in caring for patients.							
Physicians depend on me as much as I depend on them.							
Physicians are willing to work with me to discuss and optimize our patients' drug therapy.							
Physicians accept my advice on our patients' drug therapy.							

Now, thinking about your contact with physicians, tick the answer that best applies to the questions below.

37. How often do you interact with physicians to clarify questions about the medications prescribed to your patients?
    - every day
    - several times a week
    - once a week
    - once a month
    - less common
    - never

38. How often do you make suggestions to physicians regarding medication use?
    - every day
    - several times a week
    - once a week
    - once a month
    - less common
    - never

Open question:
What would improve collaboration between pharmacists and family doctors? All ideas are welcome!

Part E

Case Vignettes

In the following there will be three case-vignettes. Imagine a GP asked you to review 3 patients' medication lists and to suggest medications suitable for stopping or reducing (deprescribing). The situation of each patient is described in a case vignette. All the information that you need to make your decisions is described in the text and it is not possible to have any further information about the patient.

You will now see the three case-vignettes, which differ in terms of the patient's level of dependency in activities of daily living and overall complexity of health problems. The differences among each case vignette are written in *italic*.

After each case vignette there will be a few questions asking you which medications you would recommend to deprescribe.

Case vignette 1:

Patient 1, 82 years old

Social history: retired carpenter, *lives with his wife in a single-family home. Patient 1 prepares his medication independently, goes grocery shopping and does other work around the house and garden. The couple do not require any help from third parties.*

General health: *in a good physical and cognitive condition. MMSE 28/30.*

Other diagnoses: chronic back pain, hypertension, non-smoker, no past history of cardiovascular events, no family history of cardiovascular events

Laboratory values: dyslipidemia (LDL 3.8mmol/l), liver and kidney function are normal (taking into account the age of the patient), normal blood count. Last systolic blood pressure measurements ranged from 130 to 140mmHg.

Daily medication intake:

- Aspirin 100 mg once daily
- Atorvastatin 40 mg once daily
- Enalapril 10 mg once daily
- Amlodipine 5 mg once daily

- Paracetamol 1 g three times a day
- Tramadol 50 mg twice daily
- Pantoprazole 20mg once daily

In this case-vignette, you consider the patient:

- to have a good physical functioning and somatic condition
- to be totally independent
- to be cognitively not impaired
- to have a low risk of cardiovascular events

➔ Which of these medicines do you think are most important to the patient's health status? Please tick all that apply.

  - Aspirin 100 mg once daily
  - Atorvastatin 40 mg once daily
  - Enalapril 10 mg once daily
  - Amlodipine 5 mg once daily
  - Paracetamol 1 g three times a day
  - Tramadol 50 mg twice daily
  - Pantoprazole 20mg once daily

➔ Which of these medicines do you think are least important to the patient's health status? Please tick all that apply.

  - Aspirin 100 mg once daily
  - Atorvastatin 40 mg once daily
  - Enalapril 10 mg once daily
  - Amlodipine 5 mg once daily
  - Paracetamol 1 g three times a day
  - Tramadol 50 mg twice daily
  - Pantoprazole 20mg once daily

➔ Would you suggest stopping or decreasing the dosage of one/several medication/s? (yes/no)

➔ (branching logic with question before) Which medication/s would you stop or decrease? Please tick all that apply.

  - Aspirin 100 mg once daily

- Atorvastatin 40 mg once daily
- Enalapril 10 mg once daily
- Amlodipine 5 mg once daily
- Paracetamol 1g three times a day
- Tramadol 50 mg twice daily
- Pantoprazole 20 mg once daily

➔ Why did you choose this medication? (free text, not mandatory)

Consider that Patient 1 now had a cardiovascular event in the past (e.g. myocardial infarction three years ago). Would you stop or reduce the dosage of one/several medication/s?

- Yes
- No

➔ Which medication/s would you suggest stopping or reducing when taking into account that Patient 1 has already had a cardiovascular event in the past (e.g. myocardial infarction three years ago)?

- Aspirin 100 mg once daily
- Atorvastatin 40 mg once daily
- Enalapril 10 mg once daily
- Amlodipine 5 mg twice daily
- Paracetamol 1g three times a day
- Tramadol 50 mg twice daily
- Pantoprazole 20 mg once daily

Case vignette 2

Patient 2, 82 years of age:

Social history: retired carpenter, *lives with his wife who is in a good physical and cognitive state. Patient 2 is becoming more and more dependent; household tasks are done by his wife. Patient 2 needs help from third parties for personal hygiene, getting dressed/undressed and preparing medication.*

General state: walking pace significantly decreased over the past year, unsteady on his legs. Increasing forgetfulness and attention deficiency in the past couple of months. MMSE 22/30.

Other diagnoses: Chronic back pain, hypertension, non-smoker, no past history of cardiovascular events, no family history of cardiovascular events

Laboratory values: Dyslipidemia (LDL 3,8mmol/l), liver and kidney function are normal (taking into account the age of the patient), normal blood count. Last systolic blood pressure measurements ranged from 130 to 140mmHG.

Daily medication intake:

Aspirin 100 mg once daily

Atorvastatin 40 mg once daily

Enalapril 10 mg once daily

Amlodipine 5 mg once daily

Paracetamol 1 g three times a day

Tramadol 50 mg twice daily

Pantoprazole 20mg once daily

In this case-vignette, you consider the patient:

- to have reduced physical functioning
- to be increasingly dependent in his daily routine
- to be cognitively moderately impaired
- to have a low risk of cardiovascular events

➔ 12. Would you deprescribe or decrease the dosage of one/several medication/s?

- o Yes
- o No

➔ 13. Which medication/s would you deprescribe or decrease?

- o Aspirin 100 mg once daily
- o Atorvastatin 40 mg once daily
- o Enalapril 10 mg once daily
- o Amlodipine 5 mg once daily
- o Paracetamol 1g three times a day
- o Tramadol 50 mg twice daily
- o Pantoprazole 20 mg once daily

➔ 14. Consider that Patient 2 now had a cardiovascular event in the past (e.g. myocardial infarction three years ago). Would you deprescribe or decrease the dosage of one/several medication/s?

- Yes
- No

➔ 15. Which medication/s would you deprescribe or decrease taking into account that Patient 2 had a cardiovascular event in the past (e.g. myocardial infarction three years ago)?

- Aspirin 100 mg once daily
- Atorvastatin 40 mg once daily
- Enalapril 10 mg once daily
- Amlodipine 5 mg twice daily
- Paracetamol 1g three times a day
- Tramadol 50 mg twice daily
- Pantoprazole 20 mg once daily

<u>Case vignette 3</u>

Patient 3, 82 years of age:

Social history: retired carpenter*, lives together with his wife in a nursing home*

General health: *Patient 3 walks very little using a walker. Needs daily support for personal hygiene and getting dressed/undressed. Lack of spatial or temporal orientation. Unintended weight loss of 8kg in the past two months. MMSE 12/30.*

Other diagnoses: Chronic back pain, hypertension (last blood pressure measurements ranged from 130 to 140mmHG, systolic), non-smoker, no family history of cardiovascular events

Laboratory values: Dyslipidemia (LDL 3,8mmol/l), liver and kidney function are normal (taking into account the age of the patient), normal blood count

Daily medication intake:
Aspirin 100 mg once daily
Atorvastatin 40 mg once daily
Enalapril 10 mg once daily
Amlodipine 5 mg once daily

Paracetamol 1 g three times a day

Tramadol 50 mg twice daily

Pantoprazole 20mg once daily

In this case-vignette, you consider the patient:

- to have strongly impaired physical functioning
- to be strongly dependent in his daily routine
- to be cognitively strongly impaired
- to have a low risk of cardiovascular events

➔ 16. Would you deprescribe or decrease the dosage of one/several medication/s?

o Yes

o No

➔ 17. Which medication/s would you deprescribe or decrease?

- Aspirin 100 mg once daily
- Atorvastatin 40 mg once daily
- Enalapril 10 mg once daily
- Amlodipine 5 mg once daily
- Paracetamol 1g three times a day
- Tramadol 50 mg twice daily
- Pantoprazole 20 mg once daily

➔ 18. Consider that Patient 3 had a cardiovascular event in the past (e.g. myocardial infarction three years ago). Would you deprescribe or decrease the dosage of one/several medication/s?

- Yes
- No

➔ 19. Which medication/s would you deprescribe or decrease taking into account that Patient 3 had a cardiovascular event in the past (e.g. myocardial infarction three years ago)?

- Aspirin 100 mg once daily
- Atorvastatin 40 mg once daily
- Enalapril 10 mg once daily
- Amlodipine 5 mg once daily

- Paracetamol 1g three times a day
- Tramadol 50 mg twice daily
- Pantoprazole 20 mg once daily

End of survey

Thank you very much for your participation in our study.

We really appreciate that you took the time to do this.
